# Caregiver Burden, Psychological Distress and Quality of Life among Informal Caregivers of Patients with Head and Neck Cancer: A Longitudinal Study

**DOI:** 10.3390/ijerph192316304

**Published:** 2022-12-05

**Authors:** Kira S. Van Hof, Arta Hoesseini, Maarten C. Dorr, Irma M. Verdonck-de Leeuw, Femke Jansen, C. René Leemans, Robert P. Takes, Chris H. J. Terhaard, Robert Jan Baatenburg de Jong, Aniel Sewnaik, Marinella P. J. Offerman

**Affiliations:** 1 Department of Otorhinolaryngology and Head and Neck Surgery, Erasmus Cancer Institute, Erasmus University Medical Center, Dr. Molewaterplein 40, 3015 GD Rotterdam, The Netherlands; 2 Department of Otorhinolaryngology and Head and Neck Surgery, Cancer Center Amsterdam, Amsterdam UMC, Vrije Universiteit Amsterdam, De Boelelaan 1117, 1081 HV Amsterdam, The Netherlands; 3 Cancer Center Amsterdam, Treatment and Quality of Life, 1081 HV Amsterdam, The Netherlands; 4 Department Clinical, Neuro and Developmental Psychology, Vrije Universiteit Amsterdam, Van der Boechorststraat 7-9, 1081 HV Amsterdam, The Netherlands; 5 Amsterdam Public Health, Mental Health, 1081 HV Amsterdam, The Netherlands; 6 Department of Otolaryngology and Head and Neck Surgery, Radboud University Medical Center, 6525 GA Nijmegen, The Netherlands; 7 Department of Radiation Oncology, University Medical Center, 3584 CX Utrecht, The Netherlands

**Keywords:** informal caregivers, caregiver burden, psychological distress, quality of life, head and neck neoplasms, patient reported outcome measures

## Abstract

(1) Background: The aim of this study was to investigate caregiver burden among informal caregivers of head and neck cancer patients, in relation to distress and quality of life (QoL), and the relationship between informal caregivers and patients. (2) Methods: Data of 234 dyads from the multicenter prospective cohort study Netherlands Quality of life and Biomedical Cohort studies in cancer was used. Caregiver burden, psychological distress, global QoL, physical and social functioning were measured from baseline until 24 months after treatment. (4) Conclusions: This prospective cohort study shows the high burden of caring for HNC patients, the impact of this burden and the interaction between caregiver and patient. We suggest that healthcare professionals include caregivers in counseling and support.

## 1. Introduction

Head and neck cancer (HNC) is a type of cancer with high mortality and morbidity rates [1,2,3]. The treatment usually consists of surgery, radiotherapy, systemic therapy or a combination of these treatment modalities [4,5]. Besides the impact of the cancer itself, the consequences of treatment often interfere with vital functions, such as breathing, swallowing and speaking [6,7]. HNC is also a psychologically distressing cancer type with negative consequences on the health-related quality of life (QoL) [8,9]. Within the process of dealing with these consequences, informal caregivers are an important source of support for HNC patients. Following the multisystem theory, patients are not standalone individuals, but part of a system of interaction with their informal caregivers [10]. Psychological distress and impaired QoL, therefore, may not only occur among patients, but also among their informal caregivers [11,12,13].

Informal caregivers, most often spouses, support cancer patients both practically and emotionally from the moment of diagnosis, during the treatment period, and during follow-up [11]. Caregiving is associated with positive outcomes, such as personal growth and an enhanced relationship with the patient [14]. At the same time, caregiving can cause physical, psychological, emotional, social or financial problems as well [12,15]. This so-called, “caregiver burden”, may lead to psychological distress and impaired QoL [16,17]. Psychological distress refers to symptoms of anxiety and depression. Spouses’ distress may be related to the prospect of losing their life companion and feelings of helplessness, which both can lead to (symptoms of) a depression disorder [12,17]. It is suggested that, compared to HNC patients, spouses report higher anxiety levels, causing reduced mental health [17,18]. A recent review stated that longitudinal research investigating the caregiver burden among caregivers of cancer patients in general is needed [19]. Up to now, there is also a paucity of longitudinal research examining the caregiver burden among the caregivers of HNC patients [18,20]. Little attention is given to the impact of HNC patients’ illness and rehabilitation on the QoL and distress of informal caregivers. Furthermore, previous research often used small sample sizes [18,20,21]. One longitudinal study investigating psychological distress in caregivers from diagnosis until 6 months after treatment reported high levels of distress and impaired QoL at baseline, which improved at 6 months follow-up [22]. However, it remains unclear how the QoL and distress of caregivers of HNC patients develop beyond the 6-month follow-up and which caregivers have a higher risk for caregiver burden. Information on the risk factors may be useful for clinical practice, in order to identify informal caregivers that may benefit from additional support.

The aim of this study was to (1) investigate caregiver burden, psychological distress and QoL in the caregivers of HNC patients from diagnosis to 2 years after treatment of HNC cancer; (2) assess the relation between caregiver burden, QoL and psychological distress in caregivers; (3) explore which demographic and clinical variables are associated with caregiver burden, psychological distress and QoL over time in caregivers; (4) assess the relationship between caregiver burden and the psychological distress of caregivers at baseline with the psychological distress and QoL of their related patients over time.

## 2. Materials and Methods

### 2.1. Study Population

This study was conducted using data from the ongoing multicenter prospective cohort study the Netherlands Quality of life and Biomedical Cohort study in HNC (NET-QUBIC) [23]. From March 2014 to June 2018, 739 HNC patients and 262 informal caregivers from five HNC centers in The Netherlands were included. Inclusion criteria for patients were: 18 years or older; diagnosis of squamous cell carcinoma of the oral cavity, oropharynx, hypopharynx, or larynx, or neck lymph node metastasis of an unknown primary tumor; intention of curative treatment; and able to write, read, and speak Dutch. Exclusion criteria were: severe psychiatric comorbidity (e.g., schizophrenia, Korsakoff’s syndrome, severe dementia) or diagnosis of lymphoma, thyroid cancer, nasopharyngeal cancer, malignancy of skin, or malignancy of salivary glands. All included patients were asked if their spouse, family member or informal caregiver wanted to participate. Informal caregivers had to be able to write, read, and speak Dutch. The study was approved by the Medical Ethical Committee of the VU University Medical Center Amsterdam (2013.301(A2018.307)-NL45051.029.13). All participants provided written informed consent. A more detailed description of NET-QUBIC can be found elsewhere [24].

### 2.2. Measures

In the present study, data of patient reported outcome measures (PROMs) at baseline (shortly after diagnosis and consultation about treatment options), 3, 6, 12 and 24 months after intervention were used. The PROMs were sent per postal mail. An electronic Case Report Form (eCRF) was built (OpenClinica) and clinical information was retrieved from medical records. Performance status was scored following the WHO performance status [25]. Comorbidity was rated using the Adult Comorbidity Evaluation-27 (ACE-27) scale [26].

Caregiver burden was measured using the validated Caregiver Reaction Assessment (CRA), a 24-item questionnaire which assesses both positive and negative reactions to caregiving [15,27]. The questionnaire consists of five subscales ranging from 0 to 5: (1) self-esteem, (2) family support, (3) financial problems, (4) problems with disrupted schedules, and (5) health problems [27]. A higher score on the self-esteem subscale indicates a positive caregiver reaction (≥4 = relevant), whereas the remaining four subscales represent negative effects of caregiving (≥3 = relevant) [28]. Different scales represented a high degree of reliability (Cronbach alpha coefficient varying from 0.62 to 0.83) [15].

Psychological distress was assessed using the 14-item Hospital Anxiety and Depression scale [29]. This questionnaire distinguishes the subdomains anxiety and depression, each consisting of 7 items rated on a scale from 0 to 3. The maximum score per domain is 21, whereas a score of ≥8 indicates relevant symptoms of anxiety or depression [30]. The HADS is a feasible and reliable instrument (Cronbach’s alpha for the subscales varied from 0.67 to 0.90) [30].

Quality of life (QoL) was measured using the subscales global QoL, physical functioning and social functioning of the 30-item European Organization for Research and Treatment of Cancer Quality of Life Questionnaire (EORTC-QLQ-C30) [31]. A higher score indicates a better functioning in the particular domain (range 0–100). The cut-off scores were based on the mean score of the general population (Global QoL < 71.2, physical functioning < 89.8, social functioning < 87.5) [32]. The EORTC-QLQ-C30 is a valid instrument, of which each subdomain can be used separately (Cronbach’s alpha coefficient for global QoL 0.89, physical functioning 0.71 and social functioning 0.77) [31].

### 2.3. Statistical Analysis

Statistical analyses were performed using statistical package R [33]. Descriptive statistics were used to describe the study population and baseline outcomes. The effect of time on the categorical CRA scales was assessed with cumulative logit mixed models (clmm). Models were adjusted for possible confounders: caregivers’ sex, age, education level and caregiver type, and patients’ tumor stage, WHO status, and comorbidity. The effect of time on HADS and EORTC QLQ-C30 subscales was assessed with linear mixed model analysis for continuous outcomes. In order to assess the relation between caregiver burden and caregivers’ HADS and EORTC QLQ-C30 subscales, baseline CRA scales were added to these models as fixed effects. Lastly, the relation between HADS of caregivers and patients was evaluated with linear mixed models for patients’ HADS and EORTC outcomes, with confounders, and caregivers’ baseline CRA and HADS outcomes as fixed effects. To address the fact that PROM data over time from the same person was correlated, we used the mixed-effects model framework for repeated measurements. Furthermore, these models allow for missing data in repeated measures. The JointAI package was used to correct for missing data in the covariates, so all available data could be used in the analyses [34]. Statistical significance was determined based on a 2-sided *p*-value of less than 0.05.

## 3. Results

### 3.1. Study Population

From the 262 caregivers, nine caregivers dropped-out before the baseline assessment for varying reasons (Appendix A), and 19 caregivers did not complete any PROMs. Eventually, 234 caregiver and patient dyads were included in the current study. The sociodemographic and clinical characteristics of the study population are shown in Table 1.

### 3.2. Caregiver Burden, Psychological Distress and QoL in Caregivers of HNC Patients from Diagnosis to 2 Years after Treatment of HNC Cancer

#### 3.2.1. Baseline

**Figure 1:**
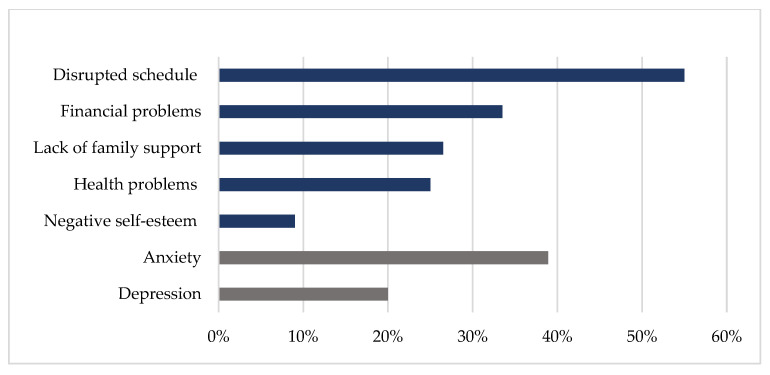
The percentage caregivers with significant relevant reactions/symptoms at baseline. For the Caregiver Reaction Assessment (CRA), a higher score on the self-esteem subscale indicates a positive caregiver reaction (≥4 = relevant), whereas the remaining four subscales represent negative effects of caregiving (≥3 = relevant). For the Hospital Anxiety and Depression Scale subdomains, a cut-off of ≥8 was used.

At baseline, 91% of the caregivers experienced positive self-esteem due to their caregiving tasks. Negative caregiver reactions were reported as well: 55% of the caregivers reported a disrupted schedule, 34% had financial problems, 27% a lack of family support, and 25% reported health problems. Clinical levels of symptoms of anxiety were reported in 39% percent of the caregivers, and 20% had symptoms of depression (Figure 1). A quarter (25%) of the caregivers reported a reduced global QoL, 30% reported a reduced physical functioning and 19% had reduced social functioning compared to the mean population.

#### 3.2.2. During Follow-Up

As shown in Table 2, self-esteem and lack of family support remained stable during follow-up. A significant decline over time of disrupted schedules, financial problems and health problems was observed (*p* < 0.001). Symptoms of anxiety and depression significantly decreased over time (*p* < 0.001) with the largest improvement in the first 6 months after treatment (anxiety: Δ = 2.9, depression: Δ = 1.9). At long-term follow-up, the mean anxiety and depression scores returned to normal population levels [35]. No significant change over time was seen on global QoL, physical, and social functioning, and mean scores were above the clinical cut-off scores at all time points (Table 2).

### 3.3. The Relation between Caregiver Burden and Psychological Distress and QoL in Caregivers

Health problems due to caregiving were significantly related with more anxiety, depression, reduced global QoL, physical and social functioning in caregivers at all measurement moments (Table 4). Self-esteem, disrupted schedule, financial problems and lack of family support at baseline were not found to be associated with psychological distress or QoL.

### 3.4. Variables Associated with Caregiver Burden, Psychological Distress and QoL over Time in Caregivers

In Table 3, the significant variables associated with caregiver burden are shown. Female gender was associated with more health problems (*p* = 0.013). Compared to caregivers with lower education, high education levels were associated with fewer financial problems (*p* < 0.001), but more problems with a disrupted schedule (*p* = 0.039). Compared to caring for a spouse, caring for a patient with “another” type of relationship was associated with reduced self-esteem (*p* = 0.007) and more problems with lack of family support (*p* = 0.017). In addition, patient characteristics were found to be associated with a higher caregiver burden: compared to tumor stage I, caring for patients with tumor stage III and IV was associated with more problems with disrupted schedules (*p* < 0.033). Caring for patients not able to carry out activities without restrictions (WHO stage I–II), was significantly associated with more problems with disrupted schedule, lack of family support compared to caregivers with patients with a WHO stage 0 (*p* < 0.05). Compared to caring for patients without comorbidity, patients’ severe comorbidity was related more with financial problems (*p* < 0.001).

In Table 4, variables associated with psychological distress and QoL are reported. Female gender was significant associated with higher anxiety levels (*p* = 0.039). Higher age was associated with reduced physical functioning (*p* < 0.001). Furthermore, higher education was associated with reduced QoL (*p* = 0.030). Caring for patients with WHO stage I–II, was significantly associated with lower QoL and reduced physical functioning compared to caregivers with patients with a WHO stage 0 (*p* < 0.05).

### 3.5. The Relationship between Caregiver Burden and Psychological Distress of Caregivers at Baseline with Psychological Distress and QoL of Their Related Patients over Time

**Figure 2:**
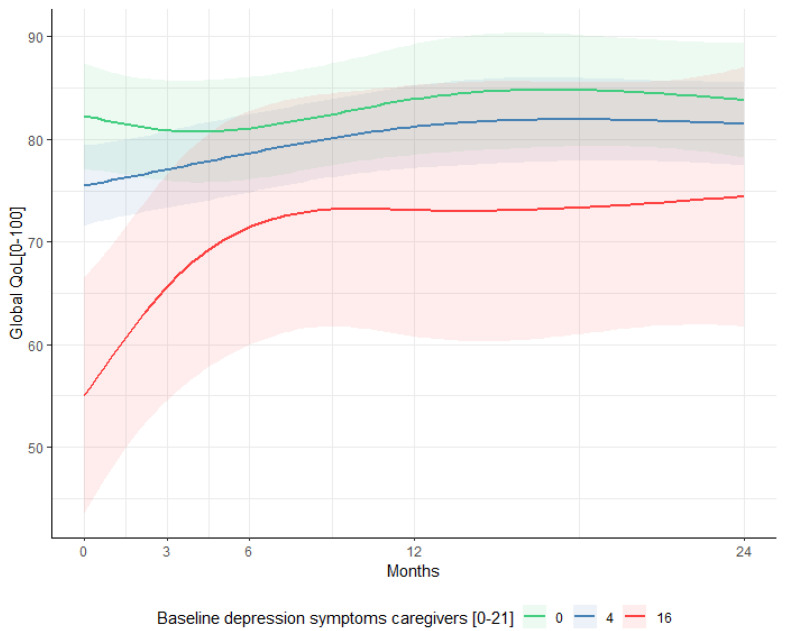
The association between depression symptoms of caregivers at baseline with global QoL of patients at all time points. The red line indicates QoL of patients over time when at baseline depression was the highest score in our cohort (HADS depression = 16), the blue line indicates the mean depression symptoms of caregivers in our cohort (HADS depression = 4), and the green line indicates no symptoms of depression (HADS depression = 0). The 95% confidence interval of the predicted anxiety and depression scores are presented in the lighter color. Outcomes were calculated with a linear mixed model, adjusted for gender, age, education level, disease stage, comorbidity and WHO status, and caregivers’ baseline Caregiver Reaction Assessment (CRA) and Hospital Anxiety and Depression Scale (HADS) outcomes.

When caregivers had higher depression levels at baseline, this was associated with a reduced global QoL in patients (*p* = 0.034) over time (Figure 2). No significant associations between the caregiver burden (CRA) at baseline and patients’ psychological distress were found.

## 4. Discussion

We aimed to investigate caregiver burden, psychological distress and quality of life (QoL) of caregivers of head and neck cancer (HNC) patients from diagnosis up to 24 months after treatment. In order to explore dyad interaction, we assessed the relationship between caregiver burden and the psychological distress of caregivers and their related patients. This study further builds on research with a cross-sectional method that examined the relationship between the psychological distress of HNC dyads [10,21,36]. To our knowledge, we reported on the largest longitudinal cohort of both HNC informal caregivers and patients in the literature. Our results are in line with earlier research that stated that patients and caregivers both have to deal with the consequences of HNC, and should be seen and approached as one unit following the multisystem theory [10,37,38,39].

The majority of the informal caregivers reported a positive impact on self-esteem due to caregiving, which continued during follow-up. However, a high percentage also reported negative consequences of caring. During the long-term follow-up, the most negative aspects of caregiving decreased significantly: disrupted schedules, financial problems and health problems (Table 2). As long-term longitudinal research on caregiver burden in HNC is lacking, it is difficult to compare our findings with existing literature [20]. Due to the significant decrease in caregiver burden over time, the timing of measurement in a cross-sectional study is essential when comparing results. Offerman et al. and Verdonck-de Leeuw et al. described caregiver burden in cross-sectional cohorts of spouses of HNC patients and reported contradictory percentages of spouses with negative or positive caregiver reactions [13,21]. However, caregiver burden was measured once after treatment with a wide range of multiple years. The few available longitudinal cohort studies measuring the caregiver burden of cancer caregivers in general also found the highest burden on the subdomain, disrupted schedule, which decreased within 6 months [40,41]. However in these cohorts, financial problems and health problems remained stable during follow-up. Comparable to our study, self-esteem remained stable as well [40,41]. When comparing our results to a longitudinal cohort of caregivers of patients with lung cancer (6 months follow-up), we found fewer financial problems and disrupted schedules, more problems with lack of family support and comparable self-esteem and health problems [39]. However, compared to a longitudinal cohort with colorectal cancer, more problems with disrupted schedule, financial problems and health problems were found in our cohort [41]. In studies comparing the caregiver burden in HNC with other types of malignancies, a higher caregiver burden was reported in HNC caregivers than in caregivers for patients with breast or ovarian carcinoma [42,43]. Besides the possible effect of cancer type, these findings may also be explained by the differences in culture of the study populations, ranging from the Netherlands, South Korea to the United States.

At baseline, elevated symptoms of distress were seen, but within six months these levels decreased to normative levels. This confirms earlier research reporting high distress in caregivers of HNC patients, and a reduction of distress during the first 6 months after treatment [13,18,44,45]. Lambert et al. analyzed anxiety and depression in varying cancer types and stated that HNC caregivers were more vulnerable than the caregivers of patients with prostate, breast, melanoma and colorectal cancer [46]. They suggested that “blaming the patient for its disease (due to lifestyle)”, persistent functional problems, and problems with patients’ body image due to mutilating interventions could be the reason for the reduced psychological function of HNC caregivers [46].

Health problems due to caregiving assesses the caregiver’s feeling that their own physical health had worsened since the start of caregiving [15]. We found that health problems at baseline were associated with psychological distress and reduced global QoL, physical and social functioning of caregivers over time. This points out that informal caregivers with a worsening of their physical health are vulnerable for reduced functioning during follow-up. Earlier research into cancer caregivers found that health problems due to caregiving were associated with psychological distress as well [39,47]. No significant relations between the other domains of caregiving and psychological distress or QoL were found in this study.

In our cohort, we found several risk factors for a high caregiver burden, psychological distress and reduced QoL in caregivers. Female gender was associated with higher anxiety levels and health problems in caregivers, which is a frequently reported predictor of psychological distress in caregivers [13,48,49,50]. Higher education levels were associated with a disrupted schedule, and fewer problems with finances. This can be explained by the busier schedules and fewer financial problems of caregivers from higher socioeconomic classes, which was found in another study as well [51]. Contrary to our expectations, higher education was also associated with a reduced global QoL. In the literature, mixed results on the influence of educational level are reported [13,18,45,52]. As expected, higher age was associated with reduced physical functioning. In accordance with the review of Longacre et al., age was not significantly associated with symptoms of anxiety and depression or QoL [16].

Caring for a patient with a higher WHO performance status (I-II) was related with reduced QoL, physical functioning, self-esteem and problems with lack of family support. Caring for patients with a higher tumor stage was associated with a disrupted schedule and health problems. Karlsson et al. did find that caring for patients with an advanced tumor stage was associated with reduced QoL and psychological distress [49]. Others reported contradictory findings as they found no relation between tumor stage and HADS scores [21,53]. However, both studies had a small sample of patients (<45 informal caregivers) and no long-term follow-up [21,53]. In our cohort, caregivers, with relations to the patient other than spouses or children, reported more problems with lack of family support and less positive self-esteem due to the caregiving. This finding does not support earlier research that found that spouses had a higher burden over time [22,40,50]. Notwithstanding, it is possible that the small size of the group of caregivers with other relationships to the patient (3.8%) had an influence on this outcome.

The current study confirms the interaction between caregivers and patients, as symptoms of depression in caregivers seem to be a risk factor for a reduced QoL in patients (Figure 2). Nonetheless, no association between the caregiver burden at baseline and patients psychological distress or QoL was found in this study. Research in HNC and cancer in general also showed that caregiver and patients are interrelated [11,51,54,55,56]. However Verdonck-de Leeuw et al., found no significant associations between spouses’ distress and the functional and social problems of HNC patients [21].

### 4.1. Strengths and Limitations

One of the major strengths is the large cohort of both informal caregivers and HNC patients that was prospectively followed until two years after treatment. In this way, all different phases of the disease trajectory were evaluated, and the long-term effects of caregiving for HNC patients could be established. Furthermore, we used a multilevel design for repeated measurements outcomes. This unique statistical design is able to assess the effect of time and risk factors, while using all available data. Although the sample of informal caregivers and patients was large, the response rate during follow-up reduced. For ethical reasons, the caregivers of HNC patients that died during follow-up automatically dropped out from that moment on. In total, 31 patients and 2 caregivers died during follow-up (Appendix A). Two years after treatment, only 136 caregivers (68% of the dyads where both caregiver and patient were alive) completed the questionnaires [57]. It is possible that dyads with more problems due to caregiving were more willing to complete the questionnaires years after treatment. Furthermore, it is possible that participating couples were not representative for the total patient population, as participation was not obligatory. Of the approached patients (n = 1861), 40% decided to participate in the NET-QUBIC cohort. In a third of the included patients (n = 262), informal caregivers were very willing to participate as well [23]. Lastly, despite the fact that we found significant differences in caregiver burden over time, we are not able to state that these differences are clinically relevant, due to the fact that currently no minimal clinically important differences (MCID) for the CRA are available [43].

### 4.2. Clinical Implications and Future Perspectives

From the moment of diagnosis until six months after treatment, especially, informal caregivers experience a high caregiver burden and psychological distress. Furthermore, symptoms of depression in caregivers at baseline seem to interfere with patients QoL over time. This underscores the importance of addressing the mental health of informal caregivers. The early assessment of risk factors and provision of information on what to expect is advocated for patients and their caregivers as both of them have to be prepared for living a life after HNC treatment. Screening for psychological problems and early referral to (psychological) support in the first line, if needed, may ensure caregivers are able to be the important source of support for patients and thus avoid the creation of “a second patient”. Knowledge of the risk factors can be used to identify those caregivers that may benefit from additional counseling and psychological support, such as the female gender or caring for patients that are not your spouse or parent, with a high WHO stage, comorbidity or severe tumor stage. Furthermore, caregivers with lower education levels seem to be more prone to financial problems and highly educated caregivers more likely to have problems with a disrupted schedule and reduced QoL. This can be explained by the fewer financial problems, but busier schedules of caregivers from higher socioeconomic classes. More research is needed to evaluate the course of symptoms of distress and QoL over time within dyads with dyadic multilevel models [58]. Furthermore, more knowledge is needed about the impact of coping and self-efficacy, and which supportive care needs the informal caregivers of HNC patients experience during all phases of the healthcare trajectory [20,59].

## 5. Conclusions

This longitudinal study on the psychological distress and quality of life (QoL) of caregivers of HNC patients identifies the high caregiver burden and its effect on QoL and psychological distress. Female gender, education level, caring for a patient with higher WHO stage, severe tumor stage or comorbidity were associated with a high caregiver burden. We found that caregivers and patients are interrelated, as depression levels of caregivers at baseline were associated with reduced QoL in patients over time. Screening for vulnerable caregivers and early referral for support may ensure that caregivers are able to be the important source of support for patients and that a “second patient” is not created. Future studies are needed to investigate which supportive care is best for this group of informal caregivers.

## Figures and Tables

**Table 1:** Descriptive characteristics of informal caregivers and patients.

	Patients (N = 234) Mean (SD) Frequency (%)	Total no. Missing (%)	Caregivers (N = 234) Mean (SD) Frequency (%)	Total no. Missing (%)
Age, years	63.6 (9.6)	0 (0%)	59.4 (11.3)	0 (0%)
Age, range	35–85	0 (0%)	19–88	0 (0%)
Gender		0 (0%)		0 (0%)
Male	177 (75.6%)		64 (27.4%)	
Female	57 (24.4%)		170 (72.7%)	
Caregiver type				
Spouse Daughter/son Other			199 (85.0%) 26 (11.1%) 9 (3.8%)	0 (0.0%)
Education level		15 (6.4%)		13 (5.5%)
Low Intermediate High	83 (35.5%) 62 (26.5%) 74 (31.6%)		82 (36.9%) 62 (27.9%) 78 (35.1%)	
Tumor site		0 (0%)		
Oral cavity Oropharynx Hypopharynx Larynx Unknown primary	68 (29.1%) 77 (32.9%) 13 (5.6%) 67 (28.6%) 9 (3.8%)			
Disease stage				
I II III IV	54 (23.1%) 43 (18.4%) 37 (15.8%) 100 (42.7%)	0 (0%)		
WHO performance		0 (0%)		
0	176 (75.2%)			
I–II	58 (24.8%)			
Comorbidity		16 (6.8%)		
None	65 (29.8%)			
Mild	85 (39.0%)			
Moderate	44 (20.2%)			
Severe	24 (11.0%)			

**Table 2:** The course over time of the primary outcome measures among caregivers.

	Clinical Cut-Off *	T0 Mean (SD)	M3 Mean (SD)	M6 Mean (SD)	M12 Mean (SD)	M24 Mean (SD)	β (95% CI)/Month	OR	*p*-Value
Caregivers		n = 234	n = 196	n = 172	n = 161	n = 136			
CRA **									
Self-esteem	≥4	4.2 (0.6)	4.2 (0.6)	4.1 (0.6)	4.1 (0.6)	4.1 (0.6)	−0.02 (−0.04 to 0.00)	0.98	0.109
Disrupted schedule	≥3	2.6 (0.8)	2.4 (0.8)	2.2 (0.9)	2.1 (0.8)	1.9 (0.8)	−0.10 (−0.13 to −0.07)	0.90	<0.001
Lack of family support	≥3	2.1 (0.7)	2.1 (0.8)	2.1 (0.8)	2.1 (0.8)	2.2 (0.8)	0.02 (−0.00 to 0.04)	1.02	0.052
Financial problems	≥3	2.2 (0.8)	1.9 (0.8)	1.9 (0.8)	1.9 (0.8)	1.7 (0.8)	−0.06 (−0.09 to −0.04)	0.94	<0.001
Health problems	≥3	2.1 (0.6)	2.1 (0.8)	2.0 (0.7)	1.8 (0.7)	1.8 (0.6)	−0.07 (−0.09 to −0.05)	0.93	<0.001
Anxiety ***	≥8	7.0 (3.8)	4.5 (3.6)	4.1 (3.2)	4.1 (3.2)	3.9 (2.7)	−0.09 (−0.11 to −0.07)	-	<0.001
Depression ***	≥8	4.4 (3.6)	2.7 (3.2)	2.5 (3.0)	2.3 (2.9)	2.1 (2.3)	−0.07 (−0.09 to −0.05)	-	<0.001
Global quality of life ***	<71.2	81.3 (14.9)	82.6 (15.1)	83.1 (15.2)	84.3 (15.5)	83.5 (15.2)	0.05 (−0.02 to 0.12)	-	0.278
Physical functioning ***	<89.8	91.3 (13.4)	91.0 (13.9)	91.1 (13.1)	91.3 (12.8)	90.5 (13.4)	−0.03 (−0.09 to 0.02)	-	0.216
Social functioning ***	<87.5	94.9 (12.1)	93.5 (15.9)	95.2 (13.2)	94.5 (12.9)	95.1 (12.4)	−0.01 (−0.06 to 0.09)	-	0.787

* Clinical cut off scores represent: clinically relevant symptoms for the Caregiver Reaction Assessment (CRA) and Hospital Anxiety and Depression Scale (HADS). For quality of life (EORTC) domains, the mean of the general population was used as cut-off score. ** The effect over time was calculated with cumulative logit mixed models; adjusted for caregivers' sex, age, education level, caregiver type, and patients' disease stage, comorbidity and WHO status. *** The mean effect over time was calculated with linear mixed models; adjusted for caregivers' sex, age, education level, caregiver type and patients' disease stage, comorbidity and WHO status, and baseline scores on CRA subscales of self-esteem, disrupted schedule, family support, financial problems and health problems.

**Table 3:** Variables that were significantly associated with caregiver burden.

Caregivers	Baseline Variable		β (95% CI)	*p*-Value
Self-esteem	Caregiver type (C)	Spouse Daughter/son	“ −0.34 (−1.71 to 1.00)	0.637
		Other	−2.56 (−4.53 to −0.66)	0.007
Disrupted schedule	WHO stage (P)	0 I–II	“ 0.717 (0.00 to 1.43)	0.050
	Tumor stage (P)	I II III IV	“ 0.75 (−0.16 to 1.69) 1.00 (0.05 to 1.93) 1.63 (0.91 to 2.42)	0.108 0.033 <0.001
	Education (C)	Low Intermediate High	“ 0.49 (−0.28 to 1.28) 0.77 (0.03 to 1.52)	0.217 0.039
Lack of family support	WHO stage (P)	0 I–II	“ 1.05 (0.25 to 1.83)	0.010
	Caregiver type (C)	Spouse Daughter/son Other	“ −0.59 (−1.94 to 0.73) 2.14 (0.41 to 3.95)	0.337 0.015
Financial problems	Comorbidity (P)	None Mild Moderate Severe	“ 0.66 (−0.17 to 1.52) 0.57 (−0.49 to 1.65) 2.20 (−0.0.96 to 3.59)	0.111 0.277 <0.001
	Education (C)	Low Intermediate High	“ −0.16 (−1.01 to 0.68) −1.30 (−2.16 to −0.41)	0.707 0.003
Health problems	Tumor stage (P)	I II III IV	“ 0.44 (−0.52 to 1.44) 0.88 (−0.11 to 1.89) 0.83 (0.05 to 1.63)	0.383 0.085 0.037
	Gender (C)	Male Female	“ 0.94 (0.20 to 1.65)	0.013

Caregiver Reaction Assessment (CRA) outcomes were calculated with a cumulative logit mixed model; adjusted for caregivers’ gender, age, education level, caregiver type and patients’ disease stage, comorbidity and WHO status. (C) stands for caregiver and (P) for patient.

**Table 4:** Variables that were significant associated with psychological distress and reduced QoL in caregivers.

Caregivers	Baseline Variable		β (95% CI)	*p*-Value
Anxiety	Gender (C)	Male Female	“ 0.93 (0.06 to 1.89)	0.039
	Health problems (C)		0.89 (0.10 to 1.67)	0.027
Depression	Health problems (C)		1.47 (0.80 to 2.14)	<0.001
Global QoL	WHO stage (P)	0 I–II	“ −6.10 (−9.99 to −2.20)	0.002
	Health problems (C)		−9.44 (−12.83 to −6.04)	<0.001
	Education (C)	Low Intermediate High	“ −1.08 (−5.38 to 3.16) −4.70 (−8.92 to −0.44)	0.621 0.030
Physical functioning	WHO stage (P)	0 I–II	“ −4.17 (−7.93 to −0.54)	0.026
	Age (C)		−0.33 (−0.49 to −0.16)	<0.001
	Health problems (C)		−8.22 (−11.37 to −4.88)	<0.001
Social functioning	Health problems (C)		−9.11 (−11.89 to −6.25)	<0.001

Hospital Anxiety and Depression Scale (HADS) and quality of life (EORTC) outcomes were calculated with a linear mixed model; adjusted for caregivers’ gender, age, education level, and patients’, disease stage, WHO status, and baseline outcomes of the caregiver reactions (self-esteem, disrupted schedule, family support, financial problems and health problems) (C) stands for caregiver and (P) for patient.

## Data Availability

Not applicable.

## References

[1] Gupta B., Johnson N.W., Kumar N. (2016). Global Epidemiology of Head and Neck Cancers: A Continuing Challenge. Oncology.

[2] Ferlay J., Soerjomataram I., Dikshit R., Eser S., Mathers C., Rebelo M., Parkin D.M., Forman D., Bray F. (2015). Cancer incidence and mortality worldwide: Sources, methods and major patterns in GLOBOCAN 2012. Int. J. Cancer.

[3] Hoesseini A., van Leeuwen N., Offerman M.P., Zhang J., Dronkers E.A., Sewnaik A., Lingsma H.F., Baatenburg de Jong R.J. (2021). Predicting survival in head and neck cancer: External validation and update of the prognostic model OncologIQ in 2189 patients. Head Neck.

[4] Hung T.M., Lin C.R., Chi Y.C., Lin C.Y., Chen E.Y.C., Kang C.J., Huang S.F., Juang Y.Y., Huang C.Y., Chang J.T.C. (2017). Body image in head and neck cancer patients treated with radiotherapy: The impact of surgical procedures. Health Qual. Life Outcomes.

[5] Richardson A.E., Broadbent E., Morton R.P. (2019). A systematic review of psychological interventions for patients with head and neck cancer. Support Care Cancer.

[6] Hutcheson K.A., Lewin J.S., Barringer D.A., Lisec A., Gunn G.B., Moore M.W., Holsinger F.C. (2012). Late dysphagia after radiotherapy-based treatment of head and neck cancer. Cancer.

[7] Beeken L., Calman F. (1994). A return to "normal eating" after curative treatment for oral cancer. What are the long-term prospects?. Eur. J. Cancer Part B Oral Oncol..

[8] Funk G.F., Karnell L.H., Christensen A.J. (2012). Long-term health-related quality of life in survivors of head and neck cancer. Arch. Otolaryngol. Head Neck Surg..

[9] Hammerlid E., Taft C. (2001). Health-related quality of life in long-term head and neck cancer survivors: A comparison with general population norms. Br. J. Cancer.

[10] Offerman M.P. (2013). Towards a Better Care for Head and Neck Cancer Patients and their Partners.

[11] Northouse L.L., Katapodi M.C., Schafenacker A.M., Weiss D. (2012). The Impact of Caregiving on the Psychological Well-Being of Family Caregivers and Cancer Patients. Seminars in Oncology Nursing.

[12] Vickery L.E., Latchford G., Hewison J., Bellew M., Feber T. (2003). The impact of head and neck cancer and facial disfigurement on the quality of life of patients and their partners. Head Neck.

[13] Offerman M.P.J., Pruyn J.F.A., De Boer M.F., Busschbach J.J.V., de Jong R.B. (2015). Psychosocial consequences for partners of patients after total laryngectomy and for the relationship between patients and partners. Oral Oncol..

[14] Li Q., Loke A.Y. (2013). The positive aspects of caregiving for cancer patients: A critical review of the literature and directions for future research. Psychooncology.

[15] Nijboer C., Triemstra M., Tempelaar R., Sanderman R., van den Bos G.A. (1999). Measuring both negative and positive reactions to giving care to cancer patients: Psychometric qualities of the Caregiver Reaction Assessment (CRA). Soc. Sci. Med..

[16] Longacre M.L., Galloway T.J., Parvanta C.F., Fang C.Y. (2015). Medical Communication-related Informational Need and Resource Preferences Among Family Caregivers for Head and Neck Cancer Patients. J. Cancer Educ..

[17] Baghi M., Wagenblast J., Hambek M., Radeloff A., Gstoettner W., Scherzed A., Spaenkuch B., Yuan J., Hornung S., Strebhardt K. (2007). Demands on caring relatives of head and neck cancer patients. Laryngoscope.

[18] Longacre M.L., Ridge J.A., Burtness B.A., Galloway T.J., Fang C.Y. (2012). Psychological functioning of caregivers for head and neck cancer patients. Oral Oncol..

[19] Kent E.E., Mollica M.A., Buckenmaier S., Smith A.W. (2019). The Characteristics of Informal Cancer Caregivers in the United States. Seminars in Oncology Nursing.

[20] Aung S.H.H., White K., Bloomfield J. (2021). The Experiences and the Needs of Caregivers of Patients with Head and Neck Cancer: An Integrative Review. Cancer Nurs..

[21] Verdonck-de Leeuw I.M., Eerenstein S.E., Van der Linden M.H., Kuik D.J., De Bree R., Leemans C.R. (2007). Distress in spouses and patients after treatment for head and neck cancer. Laryngoscope.

[22] Lee Y., Lin P.Y., Chien C.Y., Fang F.M., Wang L.J. (2018). A comparison of psychological well-being and quality of life between spouse and non-spouse caregivers in patients with head and neck cancer: A 6-month follow-up study. Neuropsychiatr. Dis. Treat..

[23] Verdonck-de Leeuw I.M., Jansen F., Brakenhoff R.H., Langendijk J.A., Takes R., Terhaard C.H.J., de Jong R.B., Smit J.H., Leemans C.R. (2019). Advancing interdisciplinary research in head and neck cancer through a multicenter longitudinal prospective cohort study: The NETherlands QUality of life and BIomedical Cohort (NET-QUBIC) data warehouse and biobank. BMC Cancer.

[24] Van Nieuwenhuizen A.J., Buffart L.M., Smit J.H., Brakenhoff R.H., Braakhuis B.J., de Bree R., Leemans C.R., Verdonck-de Leeuw I.M. (2014). A comprehensive assessment protocol including patient reported outcomes, physical tests, and biological sampling in newly diagnosed patients with head and neck cancer: Is it feasible?. Support Care Cancer.

[25] Oken M.M., Creech R.H., Tormey D.C., Horton J., Davis T.E., McFadden E.T., Carbone P.P. (1982). Toxicity and response criteria of the Eastern Cooperative Oncology Group. Am. J. Clin. Oncol..

[26] Piccirillo J.F., Tierney R.M., Costas I., Grove L., Spitznagel E.L. (2004). Prognostic Importance of Comorbidity in a Hospital-Based Cancer Registry. JAMA.

[27] Given C.W., Given B., Stommel M., Collins C., King S., Franklin S. (1992). The caregiver reaction assessment (CRA) for caregivers to persons with chronic physical and mental impairments. Res. Nurs. Health.

[28] Sandstedt P., Littorin S., Cröde Widsell G., Johansson S., Gottberg K., Ytterberg C., Olsson M., Widén Holmqvist L., Kierkegaard M. (2018). Caregiver experience, health-related quality of life and life satisfaction among informal caregivers to patients with amyotrophic lateral sclerosis: A cross-sectional study. J. Clin. Nurs..

[29] Zigmond A.S., Snaith R.P. (1983). The hospital anxiety and depression scale. Acta Psychiatr. Scand..

[30] Bjelland I., Dahl A.A., Haug T.T., Neckelmann D. (2002). The validity of the Hospital Anxiety and Depression Scale. An updated literature review. J. Psychosom. Res..

[31] Aaronson N.K., Ahmedzai S., Bergman B., Bullinger M., Cull A., Duez N.J., Filiberti A., Flechtner H., Fleishman S.B., de Haes J.C. (1993). The European Organization for Research and Treatment of Cancer QLQ-C30: A quality-of-life instrument for use in international clinical trials in oncology. J. Natl. Cancer Inst..

[32] Scott N. (2008). EORTC-QLQ-C30 Reference Values.

[33] R Core Team (2020). R: A Language and Environment for Statistical Computing. R Foundation for Statistical Computing, Vienna, Austria. https://www.R-project.org/.

[34] Erler N.S., Rizopoulos D., Lesaffre E.M.E.H. (2019). JointAI: Joint Analysis and Imputation of Incomplete Data in R. arXiv.

[35] Hinz A., Brähler E. (2011). Normative values for the hospital anxiety and depression scale (HADS) in the general German population. J. Psychosom. Res..

[36] Hodges L.J., Humphris G.M. (2009). Fear of recurrence and psychological distress in head and neck cancer patients and their carers. Psychooncology.

[37] Bronfenbrenner U. (1979). The Ecology of Human Development: Experiments by Nature and Design.

[38] Naar-King S., Ellis D., Kolmodin K., Cunningham P., Jen K.L.C., Saelens B., Brogan K. (2009). A randomized pilot study of multisystemic therapy targeting obesity in African-American adolescents. J. Adolesc. Health.

[39] Milbury K., Badr H., Fossella F., Pisters K.M., Carmack C.L. (2013). Longitudinal associations between caregiver burden and patient and spouse distress in couples coping with lung cancer. Support Care Cancer.

[40] La I.S., Johantgen M., Storr C.L., Zhu S., Cagle J.G., Ross A. (2021). Caregiver burden and related factors during active cancer treatment: A latent growth curve analysis. Eur. J. Oncol. Nurs..

[41] Nijboer C., Triemstra M., Tempelaar R., Mulder M., Sanderman R., van den Bos G.A. (2000). Patterns of caregiver experiences among partners of cancer patients. Gerontologist.

[42] Astrup G.L., Hofsø K., Bjordal K., Rustøen T. (2020). Cancer patients' diagnosis and symptoms and their family caregivers' self-efficacy and social support are associated with different caregiver reactions. Eur. J. Cancer Care.

[43] Langenberg S.M.C.H. (2021). Caring for a Patient with Cancer—The Psychosocial Impact on Informal Caregivers. Doctoral Dissertation.

[44] Lee C.Y., Lee Y., Wang L.J., Chien C.Y., Fang F.M., Lin P.Y. (2017). Depression, anxiety, quality of life, and predictors of depressive disorders in caregivers of patients with head and neck cancer: A six-month follow-up study. J. Psychosom. Res..

[45] Rigoni L., Bruhn R.F., De Cicco R., Kanda J.L., Matos L.L. (2016). Quality of life impairment in patients with head and neck cancer and their caregivers: A comparative study. Braz. J. Otorhinolaryngol..

[46] Lambert S.D., Girgis A., Lecathelinais C., Stacey F. (2013). Walking a mile in their shoes: Anxiety and depression among partners and caregivers of cancer survivors at 6 and 12 months post-diagnosis. Support Care Cancer.

[47] Nijboer C., Triemstra M., Tempelaar R., Sanderman R., van den Bos G.A. (1999). Determinants of caregiving experiences and mental health of partners of cancer patients. Cancer.

[48] Hanly P., Maguire R., Balfe M., Hyland P., Timmons A., O’Sullivan E., Butow P., Sharp L. (2016). Burden and happiness in head and neck cancer carers: The role of supportive care needs. Support Care Cancer.

[49] Karlsson T., Johansson M., Finizia C. (2020). Well-Being of Caregivers of Patients with Laryngeal Cancer Treated by Radiotherapy. Int. Arch. Otorhinolaryngol..

[50] Langenberg S.M., van Herpen C.M., van Opstal C., Wymenga A.N., van der Graaf W.T., Prins J.B. (2019). Caregivers' burden and fatigue during and after patients' treatment with concomitant chemoradiotherapy for locally advanced head and neck cancer: A prospective, observational pilot study. Support Care Cancer.

[51] Utne I., Miaskowski C., Paul S.M., Rustøen T. (2013). Association between hope and burden reported by family caregivers of patients with advanced cancer. Support Care Cancer.

[52] Ross S., Mosher C.E., Ronis-Tobin V., Hermele S., Ostroff J.S. (2010). Psychosocial adjustment of family caregivers of head and neck cancer survivors. Support Care Cancer.

[53] Badr H., Gupta V., Sikora A., Posner M. (2014). Psychological distress in patients and caregivers over the course of radiotherapy for head and neck cancer. Oral Oncol..

[54] Hagedoorn M., Sanderman R., Bolks H.N., Tuinstra J., Coyne J.C. (2008). Distress in couples coping with cancer: A meta-analysis and critical review of role and gender effects. Psychol. Bull..

[55] Hodges L.J., Humphris G.M., Macfarlane G. (2005). A meta-analytic investigation of the relationship between the psychological distress of cancer patients and their carers. Soc. Sci. Med..

[56] Hodgkinson K., Butow P., Hunt G.E., Wyse R., Hobbs K.M., Wain G. (2007). Life after cancer: Couples' and partners' psychological adjustment and supportive care needs. Support Care Cancer.

[57] Jansen F., Brakenhoff R.H., Baatenburg de Jong R.J., Langendijk J.A., Leemans C.R., Takes R.P., Terhaard C.H., Smit J.H., Verdonck-de Leeuw I.M. (2022). Study retention and attrition in a longitudinal cohort study including patient-reported outcomes, fieldwork and biobank samples: Results of the Netherlands quality of life and Biomedical cohort study (NET-QUBIC) among 739 head and neck cancer patients and 262 informal caregivers. BMC Med. Res. Methodol..

[58] Gutiérrez-Colina A.M., Lee J.L., VanDellen M., Mertens A., Marchak J.G. (2017). Family Functioning and Depressive Symptoms in Adolescent and Young Adult Cancer Survivors and Their Families: A Dyadic Analytic Approach. J. Pediatr. Psychol..

[59] Wang T., Mazanec S.R., Voss J.G. (2021). Needs of Informal Caregivers of Patients with Head and Neck Cancer: A Systematic Review. Oncol. Nurs. Forum..

